# Pulmonary Tumor Thrombotic Microangiopathy in a Patient With Male Breast Cancer: A Report of a Rare Case

**DOI:** 10.7759/cureus.103616

**Published:** 2026-02-14

**Authors:** Keiichi Miyashita, Yoshiaki Ito, Hideki Kaneko, Kentaro Nabata, Akiko Kawasumi, Yuya Murata, Akira Matsui, Shigeo Okuda

**Affiliations:** 1 Diagnostic Radiology, National Hospital Organization Tokyo Medical Center, Tokyo, JPN; 2 Pathology, National Hospital Organization Tokyo Medical Center, Tokyo, JPN; 3 Breast Surgery, National Hospital Organization Tokyo Medical Center, Tokyo, JPN

**Keywords:** breast cancer male, ct pulmonary findings, male breast neoplasm, pulmonary hypertension acute, pulmonary microangiopathy, pulmonary tumor thrombotic microangiopathy, respiratory failure acute, tumor embolism pulmonary, vascular tumor microthrombi, venous tumor thrombus

## Abstract

Pulmonary tumor thrombotic microangiopathy (PTTM) is a rare but serious complication that can occur in patients with advanced cancer. It often leads to rapidly worsening respiratory failure and right heart failure and is typically diagnosed only postmortem. While PTTM has been reported in various cancers, it is extremely rare in male patients with breast cancer, which is itself an uncommon malignancy. We report the case of a man with hormone receptor-positive breast cancer who underwent surgery followed by adjuvant chemotherapy. Several months later, he developed progressive dyspnea and increased levels of tumor markers. Although contrast-enhanced computed tomography (CT) scans revealed no evidence of pulmonary thromboembolism, the patient's right heart failure rapidly worsened, and he died shortly after hospital admission. Autopsy revealed classic histopathological features of PTTM, including tumor emboli and fibrous intimal thickening in the pulmonary arteries. To our knowledge, this is the first English-language report of PTTM arising from male breast cancer. This case highlights the importance of considering PTTM in patients with rare malignancies such as male breast cancer, particularly when they present with unexplained respiratory deterioration and signs of pulmonary hypertension. Early clinical suspicion may help guide timely therapeutic decisions.

## Introduction

Pulmonary tumor thrombotic microangiopathy (PTTM) was first described as a distinct clinicopathological condition in 1990 [1]. It is a rare and highly lethal complication of malignancy characterized by tumor embolization within the pulmonary vasculature and activation of the coagulation cascade, leading to progressive pulmonary hypertension, acute hypoxemic respiratory failure, and right heart failure, often culminating in death within days [2]. In the clinical setting, the true incidence of PTTM remains uncertain because most cases are diagnosed postmortem. In an autopsy-based series, 21 of 630 carcinoma cases (3.3%) were diagnosed with PTTM [1], suggesting that it is an uncommon but likely under-recognized complication of advanced malignancy. Histologically, PTTM is defined by widespread microscopic tumor emboli accompanied by fibrocellular intimal proliferation and progressive luminal narrowing of small pulmonary arteries, thereby distinguishing it from more common entities such as pulmonary thromboembolism (PTE) [3].

PTTM is most commonly associated with gastric cancer, which accounts for approximately 59% of reported cases. Other primary tumors include breast cancer (approximately 10%) and lung cancer (approximately 6%). The majority of PTTM cases arise from adenocarcinomas [2].

Although breast cancer is a recognized primary site of PTTM, nearly all reported cases involve female patients. PTTM arising from male breast cancer is extremely rare and is largely absent from the English literature. The scarcity of reports on male patients likely mirrors the low incidence of male breast cancer. However, given the extreme lethality of PTTM and the limited number of documented cases of male breast cancer, reporting additional cases is valuable to expand the clinical literature and reinforce awareness that PTTM can occur in breast cancer patients irrespective of sex.

## Case presentation

**Figure 1:**
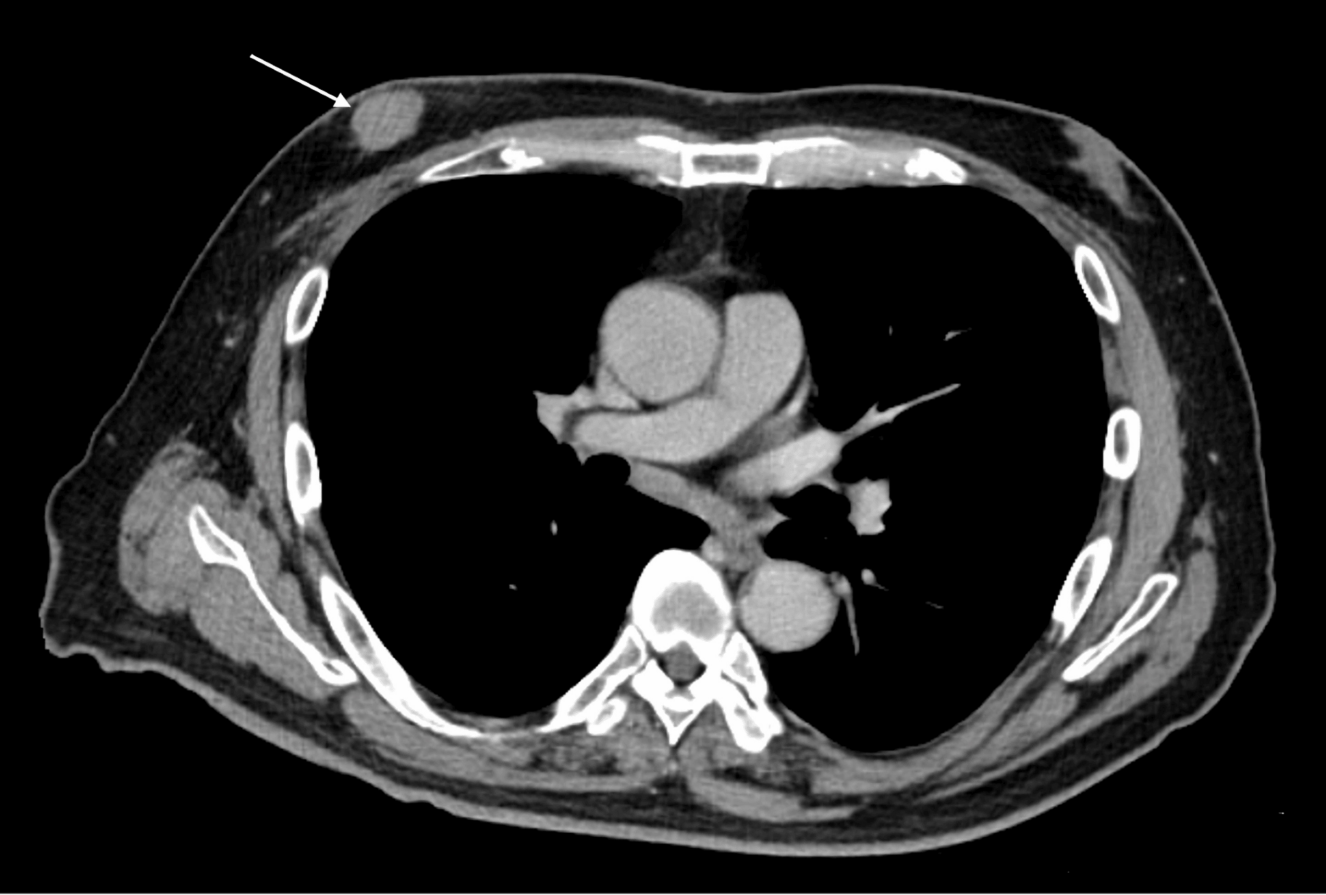
Contrast-enhanced axial chest computed tomography on initial presentation Contrast-enhanced axial chest computed tomography image showing a well-defined, homogeneously enhancing 2.3-cm mass located immediately beneath the right nipple, representing the primary breast tumor.

A man in his 70s presented to our hospital in April 2023 with a chief complaint of a right nipple mass (Figure 1). A core needle biopsy confirmed the diagnosis of invasive ductal carcinoma. His past medical history revealed lifestyle-related diseases, including angina pectoris and diabetes; however, he had no family history of breast or ovarian cancer.

In June 2023, he underwent a right mastectomy with axillary lymph node dissection. Postoperative pathological diagnosis revealed luminal B-type invasive ductal carcinoma, which was staged as pT4bN1miM0, pStage IIIB (American Joint Committee on Cancer (AJCC) classification). Given the high risk of recurrence, adjuvant chemotherapy was initiated postoperatively. The treatment plan consisted of four cycles of fluorouracil, epirubicin, and cyclophosphamide (FEC) followed by four cycles of docetaxel (DTX). At the time of disease progression, the patient had completed three cycles of DTX and was still receiving adjuvant chemotherapy.

**Figure 2:**
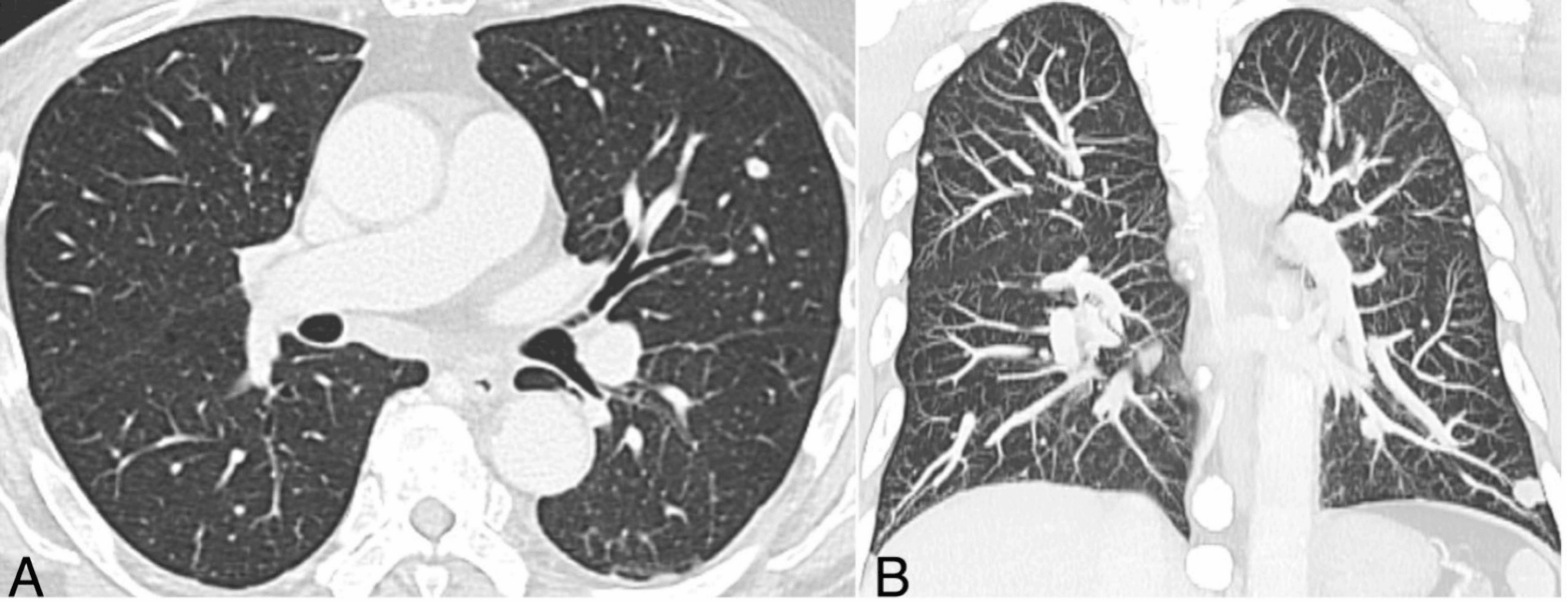
Chest computed tomography demonstrating multiple pulmonary nodules Chest computed tomography images. (A) Axial view and (B) coronal maximum intensity projection image demonstrating multiple randomly distributed pulmonary nodules in both lungs, with interval increases in number and size compared to the previous examination, suggestive of pulmonary metastases.

Eight months after surgery, elevated serum tumor markers (CA15-3: 75.5 U/mL; CEA: 9.8 ng/mL) were noted after the completion of three cycles of DTX. As recurrence or progression during adjuvant therapy is relatively uncommon in breast cancer, postoperative computed tomography (CT) imaging was planned after the completion of the fourth DTX cycle. However, approximately one month later, the patient developed acute respiratory deterioration before scheduled imaging could be performed. Contrast-enhanced CT revealed no evidence of PTE; however, thrombocytopenia and a markedly elevated D-dimer level raised suspicion of disseminated intravascular coagulation (DIC), prompting hospital admission and the initiation of heparin therapy. Pulmonary nodules present prior to surgery had increased in size, suggesting disease progression with pulmonary metastasis (Figure 2).

**Figure 3:**
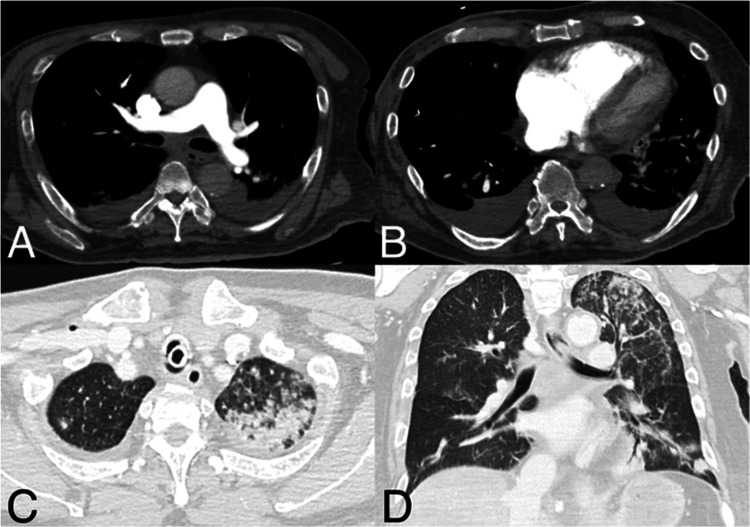
Contrast-enhanced computed tomography on the third day of hospitalization Contrast-enhanced computed tomography findings. (A) No pulmonary thromboembolism is observed in the pulmonary arteries. (B) Right ventricular enlargement, contrast reflux into the inferior vena cava, and bilateral pleural effusions are present. Periportal collar signs and subserosal edema of the gallbladder (not shown) are noted, suggesting acute right heart failure secondary to right ventricular overload. (C, D) Centrilobular nodular opacities, consolidation, and interlobular septal thickening were observed in the left upper lobe, and inflammatory changes and carcinomatous lymphangitis were considered in the differential diagnosis.

On the third day of hospitalization, the patient developed sudden respiratory distress, culminating in cardiopulmonary arrest. Return of spontaneous circulation (ROSC) was achieved after resuscitation efforts. Transthoracic echocardiography (TTE) revealed marked right ventricular strain. Follow-up contrast-enhanced CT ruled out PE but revealed right ventricular enlargement, contrast reflux into the inferior vena cava, a periportal collar sign, edematous thickening of the gallbladder wall, and bilateral pleural effusions, without evidence of pericardial effusion, indicating acute right heart failure secondary to right ventricular overload (Figure 3).

Additionally, centrilobular nodular opacities, consolidation, and interlobular septal thickening were observed in the left upper lobe, with inflammatory changes and carcinomatous lymphangitis considered in the differential diagnosis (Figure 3). Despite intensive care, the patient's respiratory and hemodynamic status deteriorated rapidly, leading to death on the same day.

**Figure 4:**
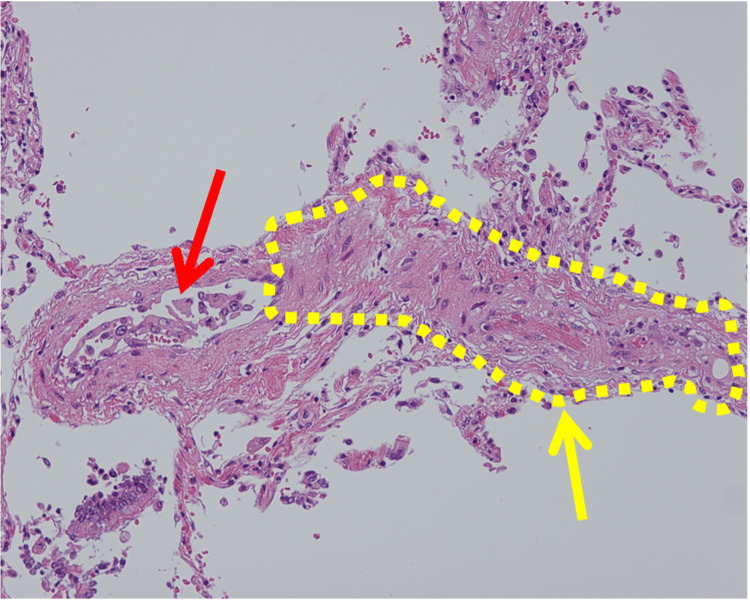
Histopathological findings of pulmonary tumor thrombotic microangiopathy Histopathological findings of the lung (H&E staining). The red arrow indicates tumor emboli within a small pulmonary artery, while the yellow arrow highlights fibrocellular intimal proliferation and fibrin thrombus formation within the vessel.

An autopsy revealed PTTM secondary to metastatic breast carcinoma. Histopathological examination revealed classic features of PTTM, including tumor emboli within small pulmonary arteries, fibrous intimal thickening, and fibrin thrombus formation (Figure 4). Further findings included carcinomatous lymphangitis and widespread metastases to the bilateral hilar and tracheobronchial lymph nodes, liver, bone marrow, small intestine, and bilateral adrenal glands. Notably, metastases to the liver, small intestine, and adrenal glands and diffusely to the bone marrow were not clearly identifiable on CT imaging.

## Discussion

While PTTM has most commonly been reported in association with gastric adenocarcinoma, breast cancer is also recognized as a frequent primary tumor site for PTTM [2,3]. However, to the best of our knowledge, no cases of PTTM arising from male breast cancer have been reported in the English-language literature.

Male breast cancer itself is an uncommon entity, accounting for less than 1% of all breast cancer cases [4]. Its rarity likely contributes to the underrepresentation of male breast cancer-associated PTTM in the literature. In addition, many PTTM cases are only diagnosed postmortem, which may further obscure the true incidence of PTTM in male breast cancer patients [2,3].

In our case, the patient presented with a rapidly progressive respiratory decline following a history of high-risk, hormone receptor-positive breast cancer treated with surgery and adjuvant chemotherapy. The clinical picture, including right ventricular overload, elevated D-dimer, and lack of radiographic evidence for conventional PTE, raised clinical suspicion for PTTM, though the diagnosis was only confirmed postmortem. Previous reports note that PTTM commonly shows a laboratory pattern of elevated D-dimer, increased lactate dehydrogenase (LDH), and thrombocytopenia [2,3].

Chest CT findings in PTTM have been reported in the literature, including ground-glass opacities (82%), small nodules (86%), mediastinal or hilar lymphadenopathy (91%), and interlobular septal thickening (81%) [2]. While these findings may occur with notable frequency, they are often mild in appearance and lack specificity, making early diagnosis extremely challenging. Moreover, such findings are not pathognomonic for PTTM and may overlap with other pulmonary pathologies, such as lymphangitic carcinomatosis or interstitial lung diseases. 

Given these limitations, additional imaging modalities have been explored to improve diagnostic yield. In particular, dual-energy CT and perfusion scintigraphy can provide functional information about regional pulmonary perfusion [5-8]. In some cases, impaired peripheral blood flow due to tumor emboli may be visualized as multiple wedge-shaped perfusion defects in the lung periphery. Although these findings are also nonspecific, they may offer supportive evidence and help raise clinical suspicion for PTTM when interpreted in the appropriate clinical context. In the present case, however, the patient's clinical condition deteriorated rapidly, and additional advanced imaging modalities were not feasible given the acute clinical course.

Although antemortem diagnosis of PTTM remains challenging, several invasive diagnostic approaches have been reported, including cytologic examination from a wedged pulmonary artery catheter and transbronchial or video-assisted thoracoscopic lung biopsy [9-11]. However, these are often contraindicated due to severe hypoxemia or pulmonary hypertension, which frequently preclude safe tissue sampling.

In clinical practice, PTTM should be suspected when a patient with a known malignancy develops rapidly progressive dyspnea, signs of pulmonary hypertension or right ventricular strain, elevated D-dimer levels with or without thrombocytopenia, and no evidence of conventional PTE on contrast-enhanced CT. The presence of nonspecific CT findings, such as small nodules, interlobular septal thickening, or ground-glass opacities, may further support this suspicion in the appropriate clinical context.

Although the prognosis of PTTM remains poor, early recognition may allow for the timely modification of systemic therapy in selected patients. Prior reports have described temporary clinical stabilization following chemotherapy or targeted agents; however, the overall outcomes remain unfavorable [6-10]. Accordingly, heightened clinical suspicion may offer a limited but potentially meaningful opportunity for therapeutic interventions.

## Conclusions

PTTM is a rare but fatal complication of advanced malignancies and remains difficult to diagnose without tissue confirmation. To our knowledge, this is the first English-language report of PTTM arising from breast cancer in males. This case highlights that PTTM may occur in patients with breast cancer, irrespective of sex. Although CT findings are often nonspecific, dual-energy CT or scintigraphy may be helpful in evaluating pulmonary perfusion, which may aid in raising the suspicion of PTTM in the appropriate clinical context.
